# Impact of Different Storage Regimes on the Levels of Physicochemical Characteristics, Especially Free Acidity in Talh (*Acacia gerrardii* Benth.) Honey

**DOI:** 10.3390/molecules27185959

**Published:** 2022-09-13

**Authors:** Hael S. A. Raweh, Ahmed Yacine Badjah-Hadj-Ahmed, Javaid Iqbal, Abdulaziz S. Alqarni

**Affiliations:** 1 Melittology Research Lab, Department of Plant Protection, College of Food and Agriculture Sciences, King Saud University, Riyadh 11451, Saudi Arabia; 2 Department of Chemistry, College of Science, King Saud University, Riyadh 11451, Saudi Arabia

**Keywords:** honey quality, storage period, storage conditions, physicochemical parameters, pollen content, monofloral honey

## Abstract

This study investigates how storage conditions (temperature and duration) may affect the physicochemical parameters, especially free acidity (FA), of Talh honey originating from *Acacia gerrardii* that have naturally high FA levels. Fresh Talh honey samples were kept at 0, 25, 35, and 45 °C, and analyzed monthly over a period of eight months. The Talh honey was monofloral with 69% *A. gerrardii* pollen content. The free acidity (FA) of freshly harvested Talh honey samples was higher (93 ± 0.3 meq/kg) than that of standard limits (≤50 meq/kg) and remained stable at 0 °C throughout the storage period. A significantly increase in FA started to occur after storage for 6 months at 25 °C (103 ± 0.2 meq/kg), 2 months at 35 °C (108 ± 0.3 meq/kg), and 1 month at 45 °C (112 ± 0.3 meq/kg). After 8 months of storage, the highest FA level was recorded at 45 °C (159 ± 0.5 meq/kg), followed by 127 ± 0.3 meq/kg at 35 °C, 105 ± 0.2 meq/kg at 25 °C, and 94 ± 0.3 meq/kg at 0 °C. It was found that 0 °C was an appropriate temperature for storing honey for long time. The electrical conductivity (EC) of fresh Talh samples (1.46 ± 0.0 mS/cm) was above the accepted limit (≤0.8 mS/cm), which was slightly increased (non-significant) throughout the storage period under all the storage temperatures. Hydroxymethylfurfural (HMF), diastase activity (DN), and reducing sugars (RSs) showed normal levels only at 0 °C and 25 °C throughout the storage period. However, HMF exceeded the standard limits after the first month at 45 °C (127 ± 9.6 mg/kg) and after the second month at 35 °C (90 ± 23.5 mg/kg), DA decreased below standard limits after the second month (5 ± 1 DN) under 45 °C and after the seventh month under 35 °C (7 ± 2 DN, and RSs decreased below 60% after 2 months under 45 °C and after 6 months at 35 °C. The physicochemical parameters (moisture content, pH, color, and sucrose) were the least affected and were within the standard range throughout the storage period under all the storage temperatures. The levels of FA and EC in fresh Talh samples were higher than the acceptable limits. The moisture content, pH, color, and sucrose content were not affected by storage conditions and remained within the acceptable limits. HMF, DA, and RSs were significantly affected by storage conditions only at 35 and 45 °C. The storage of honey at low temperatures (0 and 25 °C) for up to eight months presented the least amount of changes in the honey, and the honey was unchanged from its fresh status. Honey storage at 35 and 45 °C resulted in significant changes. It is recommended that Talh honey, which normally has high acidity levels, should be stored at temperatures not exceeding 25 °C.

## 1. Introduction

Honey consumers are always concerned about honey quality due to its energetic and nutritive effects. Honey is a prominent source of energy, enriched with a high quantity of carbohydrates (80 g, with 40 g fructose, 35 g glucose, and 5 g sucrose, in 100 g of honey) and approximately 20 g of water. It contains over 180 different substances, especially organic acids (acetic acid and gluconic acid), as minor constituents that contribute substantially to the characteristic taste and acidity of honey [1]. Vitamins and minerals are present in trace amounts [2]. The quality of honey is determined by its sensorial (smell, taste, appearance, and consistency), chemical, and physical characteristics (fructose, glucose, sucrose, moisture, water insolubility, electrical conductivity, free acidity (FA), hydroxymethylfurfural (HMF), diastase activity, and ash content) [3]. These quality parameters can be negatively influenced with different factors, such as overfeeding of bees with sucrose, harvesting before ripeness, storage conditions, etc. Furthermore, fraudulent practices or the application of severe heat treatments for liquefaction or pasteurization purposes can also affect honey quality [4,5,6].

The levels of acidity and pH specify the ripeness of honey and any changes in honey quality during storage. The levels of diastase activity and HMF content reveal facts about honey purity, storage, and thermal treatment [7]. Thermal treatment at high temperature and for a prolonged duration destroys vitamins and bionutrients and leads to a rise in HMF content and, simultaneously, a decrease in diastase activity [8]. Numerous studies around the globe have investigated the impacts of temperature and storage duration on honey quality parameters in diversified conditions [8,9,10,11,12,13,14,15].

Honey extracted from acacia is locally named as Talh honey in Saudi Arabia. Previously described physicochemical parameters of Talh honey (pH: 4.18–4.46, water content: 16.52–17.32%, and total soluble solids: 80–81%) are within the range of international standards, except for FA (120–134 meq/kg) and HMF (26–168 mg/kg) content [16]. Most importantly, the FA values of Talh honey are naturally exceeding the permitted level (≤ 50 meq/kg), which undoubtedly influences its quality evaluations, consumer interest, and honey producer qualifications. A recent study by our laboratory explored the reason for high FA in Talh honey and tested acidity levels from the leaves and flowers of *Acacia gerrardii*, the contents of bee stomachs, processed honey inside beehives, and harvested honey during the flowering season of *A. gerrardii*. It was found that the high acidity levels in Talh honey were attributable to the plant origin but not to quality loss, and we recommended that quality standards for acacia honey should be modified accordingly [17].

The naturally higher FA value in Talh honey compared to standard limits is a big constraint in the marketing of Talh honey because honey experts do not accept honey with high FA. Furthermore, beekeepers face difficulty in exporting Talh honey because purchasers usually refuse to buy Talh honey after observing the high FA during honey analyses. However, it is a fact that the high FA level in Talh honey is natural, without any human manipulation. In Saudi Arabia, there is no comprehensive database characterizing Saudi Arabian honeys, which may contribute, if available, positively to reformulating a proper and nationally accepted honey quality standard for Talh honey.

The current study evaluates how temperature and storage conditions affect the physiochemical parameters and, especially, the naturally occurring high level of FA in Talh honey. These findings may assist beekeepers in maintaining their product’s nature and freshness for better marketing.

## 2. Results

### 2.1. Melissopalynological Analysis

The microscopic melissopalynological examination revealed the presence of Talh (*Acacia gerrardii*) pollen (very frequent) in the honey collected from the three regions. The percentages of pollen grains in Talh honey from the Riyadh region ranged from 82% to 93%, with an average of 88%; those from the Asir region ranged from 62% to 66% (average of 64%); and those from the Hail region ranged from 53% to 55% (average of 54%), with an overall average of 69% Talh pollen content (Table 1). The pollen density also revealed the total pollen types present in the Talh honey collected from Hail (seventeen), Riyadh (six), and Asir (nine). All the tested honey samples (Table 1) were monofloral Talh honey originating from *Acacia gerrardii* according to the approach of Louveau et al. [18] and Crane [19], i.e., the presence of at least 45% pollens of the total pollens in honey is referred to as monoforal honey. Therefore, the honey samples under investigation were confirmed as one honey type of *A. gerrardii*.

### 2.2. Physicochemical Characteristics of Honey

This study highlights the physicochemical properties of monofloral Talh honey collected from three regions in Saudi Arabia (Riyadh, Asir, and Hail) and their progressions over eight months under different temperatures of 0, 25, 35, and 45 °C. Table 2 and Table 3 showed the mean values of the physicochemical characteristics of the tested honey samples. Comparisons of the physiochemical properties of Talh honey samples collected from three different individual locations (Riyadh, Asir, and Hail) are given in Supplementary Tables S1, S3–S5.

#### 2.2.1. Free Acidity (FA)

The free acidity values of the Talh honey samples for each of the storage temperatures and storage periods showed statistically significant differences (*p* < 0.05) (Table 2). The FA values in all the collected fresh samples were >50 meq/kg, exceeding the limits of the Codex Alimentarius standard, 2001 [13] and GSO, 2014 [20]. In this respect, a vast increase in the free acidity of Talh honey was evident during the storage period, especially at 35 and 45 °C. Based on our findings, in this study, the storage temperature was found to be the most significant influencing factor on the free acidity values (*p* < 0.05). The mean values of free acidity for the tested Talh honey samples ranged from 93 ± 0.3 in fresh honey to 159 ± 0.5 meq/kg in honey stored at 45 °C meq/kg after eight months of storage (Table 2). At the end of the storage period, significant (*p* < 0.05) increases of 105 ± 0.2, 127 ± 0.3, and 159 ± 0.5 were observed in the average free acidity values in Talh honey at 25, 35, and 45 °C, respectively. However, there was no significant increase at 0 °C. At 25 °C, a significant increase started from the sixth month, whereas at 35 °C, a significant increase started from the second month. At 45 °C, a significant increase started from the first month (Table 2). Although all the values of free acidity in this study were beyond the standard limit, the results indicated that the low temperatures (0–25 °C) maintained the stability of the free acidity of Talh honey for up to 6 months without significant effects. In this study, FA had a positive and high correlation (*p* < 0.05) with storage period (0.401), storage temperature (0.631), HMF (0.852), color (0.541), moisture (0.440), and EC (0.155). In contrast, FA was negatively correlated (*p* < 0.05) with DN (−0.309), pH (−0.851), fructose (−0.821), glucose (−0.892), and sucrose (−0.422) (Table 4). Thus, low pH, DN, and sugars led to higher FA and vice versa, whereas the level of FA was directly affected by storage period, temperature, and HMF.

#### 2.2.2. Moisture Content

Table 2 showed that the moisture content values of all the honey samples at all the tested temperatures were similar during the storage period. The initial moisture mean value for fresh Talh honey was 15 ± 0.1%. During the storage period, the moisture content values of all the honey samples fluctuated between 14.7 ± 0.2 and 15.1 ± 0.1%. After 8 months of storage, the moisture content values for all the storage periods were 14.9 ± 0.1% at 0 °C, 14.7 ± 0.1% at 25 °C, 15 ± 0.1% at 35 °C, and 15.1 ± 0.1% at 45 °C. The moisture contents of Talh honey were slightly non-significantly decreased with the duration of the storage period only at 0 °C and 25 °C. The correlation analysis for moisture content showed that it was significantly positively correlated (*p* < 0.05) with EC, HMF, FA, and DN. However, it was negatively correlated (*p* < 0.05) with storage period, pH, fructose, glucose, and sucrose (Table 4).

#### 2.2.3. Color

According to the Pfund scale given in USDA, 1985 [21], all the honey samples were dark amber in this study, showing a gradual increase in color value with time and temperature. The color values varied from 123 ± 7.0 in fresh samples (dark amber) to 150 ± 0.0 (dark amber) across all the temperatures and storage periods (Table 2). The mean color values of honey after eight months of storage were 137 ± 4.0 at 0 °C and 150 ± 0.0 at 25, 35, and 45 °C, as shown in Table 2. Overall, there were significant differences (*p* < 0.05) in the colors of Talh honey according to storage temperature. There were no significant differences between months at 0 °C, whereas there were significant differences in the fresh sample colors starting after the first month (35 °C and 45 °C) and after the fourth month (25 °C). All these changes were within the range of dark amber. The color of Talh honey had a significant positive correlation with storage period, storage temperature, HMF, and FA and a significant negative correlation with moisture, pH, EC, DN, fructose, and glucose (Table 4).

**Table 2:** Physicochemical analysis of Talh *Acacia gerrardii* honey according to different temperatures and storage intervals for eight months from 2019–2020.

Parameters: (Codex Standards)	Temp. (°C)	Fresh Honey	Storage Interval (8 Months)								
			1	2	3	4	5	6	7	8	Mean
Moisture (≤20%)	0	15± 0.1 a	15 ± 0.1 a	15 ± 0.1 a	15 ± 0.1 a	15 ± 0.1 a	14.9 ± 0.1 a	14.9 ± 0.1 a	14.9 ± 0.1 a	14.9 ± 0.1 a	15 ± 0.1 AB
	25	15 ± 0.1 a	14.8 ± 0.3 a	14.8 ± 0.2 a	14.8 ± 0.2 a	14.7 ± 0.2 a	14.7 ± 0.2 a	14.7 ± 0.2 a	14.7 ± 0.2 a	14.7 ± 0.2 a	14.8 ± 0.1 C
	35	15 ± 0.1 a	14.9 ± 0.1 a	14.8 ± 0.2 a	14.9 ± 0.2 a	14.9 ± 0.2 a	15 ± 0.2 a	15 ± 0.2 a	15 ± 0.2 a	15 ± 0.2 a	14.9 ± 0.1 B
	45	15 ± 0.1 a	15 ± 0.1 a	15 ± 0.1 a	15.1 ± 0.1 a	15.1 ± 0.1 a	15.1 ± 0.1 a	15.1 ± 0.1 a	15.1 ± 0.1 a	15.1 ± 0.1 a	15.1 ± 0.1 A
Pfund Color (0–150 mm) *	0	123 ± 7.0 a	125 ± 7.0 a	126 ± 6.0 a	126 ± 6.0 a	129 ± 6.0 a	133 ± 5.0 a	136 ± 4.0 a	136 ± 4.0 a	137 ± 4.0 a	130 ± 5.0 A
	25	123 ± 7.0 e	127 ± 6.0 de	133 ± 5.0 cde	135 ± 4.0 cde	137 ± 3.0 bcd	142 ± 2.0 abc	145 ± 2.0 abc	148 ± 1.0 ab	150 ± 0.0 a	138 ± 5.0 A
	35	123 ± 7.0 b	148 ± 1.0 a	150 ± 0.0 a	150 ± 0.0 a	150 ± 0.0 a	150 ± 0.0 a	150 ± 0.0 a	150 ± 0.0 a	150 ± 0.0 a	147 ± 4.0 B
	45	123 ± 7.0 b	150 ± 1.0 a	150 ± 0.0 a	150 ± 4.0 a	150 ± 0.0 a	150 ± 0.0 a	150 ± 0.0 a	150 ± 0.0 a	150 ± 0.0 a	147 ± 4.0 C
EC (mS/cm) (≤0.8)	0	1.46 ± 0.0 a	1.52 ± 0.0 a	1.52 ± 0.0 a	1.51 ± 0.0 a	1.59 ± 0.1 a	1.61 ± 0.1 a	1.63 ± 0.1 a	1.63 ± 0.1 a	1.63 ± 0.1 a	1.57 ± 0.1 A
	25	1.46 ± 0.0 a	1.54 ± 0.1 a	1.54 ± 0.1 a	1.53 ± 0.1 a	1.63 ± 0.1 a	1.62 ± 0.1 a	1.63 ± 0.1 a	1.63 ± 0.1 a	1.63 ± 0.1 a	1.58 ± 0.1 A
	35	1.46 ± 0.0 a	1.53 ± 0.1 a	1.53 ± 0.1 a	1.52 ± 0.1 a	1.61 ± 0.1 a	1.60 ± 0.1 a	1.62 ± 0.1 a	1.62 ± 0.1 a	1.63 ± 0.1 a	1.57 ± 0.1 A
	45	1.46 ± 00 a	1.57 ± 0.1 a	1.54 ± 0.1 a	1.50 ± 0.1 a	1.60 ± 0.1 a	1.62 ± 0.1 a	1.64 ± 0.1 a	1.64 ± 0.1 a	1.64 ± 0.1 a	1.58 ± 0.1 A
pH (3.4–6.1)	0	5.1 ± 0.1 a	5.0 ± 0.1 a	5.0 ± 0.1 a	4.9 ± 0.1 a	4.9 ± 0.1 a	4.9 ± 0.1 a	4.9 ± 0.1 a	4.9 ± 0.1 a	4.9 ± 0.1 a	4.9 ± 0.1 A
	25	5.1 ± 0.1 a	4.9 ± 0.1 b	4.9 ± 0.1 b	4.9 ± 0.1 b	4.9 ± 0.1 b	4.9 ± 0.1 b	4.9 ± 0.1 b	4.8 ± 0.1 b	4.8 ± 0.1 b	4.9 ± 0.1 A
	35	5.1 ± 0.1 a	4.9 ± 0.0 b	4.8 ± 0.0 bc	4.7 ± 0.0 cd	4.7 ± 0.0 cd	4.6 ± 0.1 de	4.5 ± 0.1 ef	4.5 ± 0.0 ef	4.4 ± 0.1 f	4.7 ± 0.1 B
	45	5.1 ± 0.1 a	4.7 ± 0.0 b	4.6 ± 0.0 b	4.5 ± 0.0 c	4.4 ± 0.0 c	4.2 ± 0.0 d	4.2 ± 0.0 d	4.1 ± 0.0 e	4.0 ± 0.0 e	4.4 ± 0.1 C
Free Acidity (meq/kg) (≤50)	0	93 ± 0.3 a	93 ± 0.3 a	94 ± 0.3 a	94 ± 0.3 a	94 ± 0.3 a	94 ± 0.3 a	94 ± 0.3 a	94 ± 0.3 a	94 ± 0.3 a	94 ± 0.3 D
	25	93 ± 0.3 c	94 ± 0.3 c	96 ± 0.3 bc	98 ± 0.3 abc	100 ± 0.2 abc	101 ± 0.2 abc	103 ± 0.2 ab	103 ± 0.2 ab	105 ± 0.2 a	99 ± 0.3 C
	35	93 ± 0.3 e	101 ± 0.3 de	108 ± 0.3 dc	113 ± 0.3 bc	115 ± 0.3 bc	120 ± 0.3 ab	122 ± 0.3 ab	125 ± 0.4 a	127 ± 0.3 a	114 ± 0.5 B
	45	93 ± 0.3 e	112 ± 0.3 d	123 ± 0.4 dc	129 ± 0.4 bc	136 ± 0.5 bc	145 ± 0.5 ab	149 ± 0.5 ab	154 ± 0.5 a	159 ± 0.5 a	133 ± 0.8 A
HMF (mg/kg) (≤40) **	0	1 ± 0.2 b	1 ± 0.1 ab	1 ± 0.2 ab	1 ± 0.2 ab	2 ± 0.3 a	2 ± 0.4 a	2 ± 0.5 a	2 ± 0.4 a	2 ± 0.4 a	2 ± 0.4 C
	25	1 ± 0.2 e	3 ± 2.0 de	4 ± 1.7 cde	7 ± 2.5b cde	9 ± 3.5 abcd	10 ± 3.8 abcd	12 ± 4.3 abc	13 ± 4.1 ab	15 ± 4.9 a	8 ± 3.0 C
	35	1 ± 0.2 f	22 ± 7.0 ef	90 ± 23.5 de	109 ± 20.7 d	149 ± 22.4 cd	190 ± 29.7 bc	235 ± 31.4 ab	256 ± 35.1 ab	281 ± 43.0 a	148 ± 40.8 B
	45	1 ± 0.2 i	127 ± 9.6 h	260 ± 21.1 g	356 ± 24.9 f	474 ± 43.5 e	595 ± 32.2 d	711 ± 21.9 c	784 ± 25.4 b	897 ± 22.5 a	467 ± 99.7 A
Diastase Activity (DN) (≥8)	0	16 ± 3.0 a	15 ± 3.0 a	15 ± 3.0 a	15 ± 3.0 a	15 ± 3.0 a	15 ± 3.0 a	15 ± 3.0 a	15 ± 3.0 a	15 ± 3.0 a	15 ± 3.0 A
	25	16 ± 3.0 a	16 ± 3.0 a	15 ± 3.0 a	14 ± 3.0 a	14 ± 3.0 a	14 ± 3.0 a	13 ± 3.0 a	13 ± 3.0 a	13 ± 2.0 a	14 ± 3.0 A
	35	16 ± 3.0 a	14 ± 3.0 ab	11 ± 2.0 abc	10 ± 2.0 abc	9 ± 2.0 abc	8 ± 2.0 bc	8 ± 2.0 bc	7 ± 2.0 bc	7 ± 2.0 c	10 ± 2.0 B
	45	16 ± 3.0 a	9 ± 2.0 b	5 ± 1.0 bc	4 ± 1.0 cd	2 ± 0.0 cd	2 ± 0.0 cd	1 ± 0.0 cd	1 ± 0.0 d	0 ± 0.0 d	5.0 ± 2.0 C

Means with the same letters are not significantly different from each other (*p* < 0.05, Duncan’s test). * Color was determined in mm on the Pfund scale according to U.S. Department of Agriculture classifications. ** in tropical regions (80 mg/kg). Lower case letters in table columns represent the comparison among values of individual physicochemical parameter of fresh honey, and honey stored at different temperature (0,25,35 and 45ºC) over a period of 1-8 months. Upper case letters in last column represent the comparison among mean values of individual physicochemical parameter after eight month’s storage of honey at four different temperatures.

#### 2.2.4. Electrical Conductivity (EC)

Electrical conductivity is closely related to the concentrations of minerals, organic acids, and proteins [22]. In our study, high EC was recorded in the fresh samples (1.46 ± 0.0 mS/cm) at more than 0.8 mS/cm, the maximum limit indicated by the Codex Alimentarius standard, 2001 [13] and GSO, 2014 [20]. The EC values of Talh honey fluctuated during the storage period between 1.46 ± 0.0 mS/cm in the fresh samples and 1.64 ± 0.1 mS/cm at 45 °C (Table 2). After eight months of storage, the mean EC values reached 1.63 ± 0.1 mS/cm at 0, 25 °C, and 35 °C and 1.64 ± 0.1 mS/cm at 45 °C. At each storage temperature, there was a slightly increasing trend from the first month without significant differences (*p* > 0.05) (Table 2). EC was positively (*p* < 0.05) correlated with storage period, HMF, and FA, while a negative (*p* > 0.05) correlation with fructose, glucose, and sucrose was detected (Table 4).

#### 2.2.5. pH

Table 2 presents the average pH values of the Talh honey. There were no significant differences (*p* > 0.05) between the months at 0 °C, while there were significant decreases at 25, 35, and 45 °C starting from the first month. From the 5.1 ± 0.1 pH values of the fresh honey samples, the pH values during all the storage periods at each temperature decreased to the lowest value of 4.0 ± 0.0 at 45 °C during the eight-month storage period. Honey storage at 45 °C was the most significant influencing factor on pH value (*p* < 0.05). The pH values of all the tested Talh honey samples were acidic. After eight months of storage, the mean pH values decreased to 4.9 ± 0.1, 4.8 ± 0.1, 4.4 ± 0.1, and 4.0 ± 0.0 at 0, 25, 35, and 45 °C, respectively. pH showed a positive (*p* < 0.05) correlation with EC, DN, fructose, glucose, and sucrose. However, a negative pH (*p* < 0.05) correlation with storage period, storage temperature, HMF, and FA was recorded (Table 4).

#### 2.2.6. HMF

The values of the initial HMF contents in all the samples of fresh honey (1 ± 0.2 mg/kg) were less than those of national and international limits (≤40 mg/kg). In general, the statistical analysis showed significant differences for HMF (*p* < 0.05) at different temperatures and storage periods (Table 2). The values of HMF increased significantly at 45 °C from the first month of the storage period and at 35 °C from the second month of the storage period, exceeding the range of the international standards. At 0 °C and 25 °C, HMF increased significantly from the fourth month; however, it remained below the maximum limits of the international standards throughout the storage period of 8 months (Table 2). The initial HMF values of fresh Talh honey (1 ± 0.2 mg/kg) were increased to a maximum of 897 ± 22.5 mg/kg at 45 °C after eight months of storage (Table 2). After 8 months of storage at each temperature (0, 25°, 35°, and 45 °C), the HMF values were 2 ± 0.4, 15 ± 4.9, 281 ± 43.0, and 897 ± 22.5 mg/kg, respectively. The mean HMF values at 0, 25°, 35°, and 45 °C were 2 ± 0.4, 8 ± 0.3, 148 ± 40.8, and 467 ± 99.7 mg/kg, respectively. HMF was found to be significantly positively correlated (*p* < 0.05) with storage period (0.391), storage temperature (0.603), and FA (0.852). In contrast, a negative correlation (*p* < 0.05) of HMF with DN (−0.559), fructose (−0.842), glucose (−0.748), and sucrose (−0.272) was recorded. Thus, the level of HMF was directly associated with storage period, storage temperature, and FA. (Table 4).

#### 2.2.7. Diastase Activity (DA)

Diastase activity indicates honey freshness and is viewed as a vital measure in predicting honey quality. The values (16 ± 3.0 DN) of the fresh samples showed that diastase activity was not subjected to high temperatures during processing, with values not less than eight according to the Schade scale [23]. The decrease in diastase activity was closely related to the increase in storage temperature at 45 °C and 35 °C compared to 0 and 25 °C. The results of the statistical analysis of the Talh honey sample diastase activity showed, overall, statistically significant differences (*p* < 0.05) with temperature and storage period (Table 2). The mean value of fresh Talh honey diastase activity before storage was 16 ± 3.0 DN, and it decreased during all the storage periods and reached 0.0 ± 0.0 DN at 45 °C (Table 2), indicating a loss in quality. Following eight months of storage, the mean values of diastase activity significantly decreased (*p* < 0.05) to 15 ± 3.0, 13 ± 2.0, 7 ± 2.0, and 0.0 ± 0.0 DN under storage temperatures of 0, 25, 35, and 45 °C, respectively. The diastase activity was diminished after the first month of honey storage at 45 °C, while at 35 °C, it diminished significantly from the fifth month. However, at 0 and 25 °C, the values of DN remained normal to the end of the storage period without significant changes. A correlation analysis showed a negative (*p* < 0.05) association for DN with storage period, storage temperature, HMF, FA, and sucrose. However, a positive (*p* < 0.05) correlation was recorded for DN with fructose and glucose (Table 4). This result means that honey quality based on diastase activity significantly decreased with longer storage times and increased temperatures, as well as high acidity and HMF values.

### 2.3. Sugars

The contents of fructose, glucose, and sucrose and their evolution throughout the eight months are shown in Table 3. The temperature and storage period of honey significantly (*p* < 0.05) affected the sugar content of Talh honey. All three sugars showed a significantly declining tendency at all the temperatures, specifically at 45 and 35 °C, after eight months of storage. However, the values of sucrose remained within the standard limits during the whole storage period at all the temperatures, whereas reducing sugars remained at normal limits only at 0 and 25 °C. Under 45 °C and 35 °C, the RSs decreased below acceptable limits after 2 months and 6 months, respectively. The average values of sucrose and reducing sugars in the fresh Talh honey were 0.31 ± 0.0% and 69.5 ± 0.3%, respectively; consistent with the legislations of the Codex Alimentarius, 2001 [13] and GSO, 2014 [20], (≥60% for reducing sugars and ≤5% for sucrose), these levels decreased, ultimately, at 45 °C to 53.5 ± 0.9% and 0.0 ± 0.0%, respectively (Table 3). The comparison of sugar contents of Talh honey samples collected from three different individual locations (Riyadh, Asir, and Hail) is shown in Supplementary Tables S2, S6–S8.

#### 2.3.1. Fructose

Table 3 shows the fructose analysis for the samples of Talh honey over the eight months of storage. Overall, significant differences (*p* < 0.05) were recorded among the different temperatures and storage periods. The mean value of fructose in fresh Talh honey before storage was 38.7 ± 0.0%; it decreased across all the temperatures during the storage period to 32.0 ± 0.0%. This decrease was clearly apparent (*p* < 0.05) from the first month of honey storage at all the temperatures. The greatest decrease occurred at the storage temperature of 45 °C, followed by those at 35 °C, 25 °C, and 0 °C to 32.3 ± 0.0%, 35.3 ± 0.0%, 36.2 ± 0.0%, and 37 ± 0.0%, respectively. Table 4 shows that there was a positive (*p* < 0.05) correlation of fructose with pH, DN, glucose, and sucrose. However, a negative (*p* < 0.05) correlation for fructose was recorded with storage period, storage temperature, color, moisture, EC, HMF, and FA.

#### 2.3.2. Glucose

The data for the Talh honey glucose content during storage over eight months are shown in Table 3. Glucose values ranged from 30.8 ± 0.0% in the fresh samples to 29.7 ± 0.0%, 27.0 ± 0.0%, 23.6 ± 1.0%, and 21.2 ± 1.0% at 0, 25, 35, and 45 °C, respectively, at the end of eight months, with significant decreases (*p* < 0.05). There was a significant decrease in the percentage of glucose from the first month of the storage period at all the temperatures, and the decrease was more obvious at 45 °C, followed by that at 35 °C, and then 25 °C. Table 4 shows a positive (*p* < 0.05) correlation for glucose content with fructose, sucrose, pH, and DN. Negative (*p* < 0.05) correlations were recorded for glucose with storage period, storage temperature, color, moisture, EC, HMF, and FA.

#### 2.3.3. Sucrose

Overall, the sucrose content of the Talh honey samples showed significant differences (*p* < 0.05) across the storage temperatures and times (Table 3). The average value of sucrose in the Talh honey was less than 5% in all the samples, and this result was consistent with the limits of the Codex Alimentarius standard, 2001 [13] and GSO, 2014 [20] (≤5%). The initial sucrose content average for Talh honey was 0.31 ± 0.0%; it fluctuated during storage to 0.76 ± 0.0% after 2 months, to 0.2 ± 0.0% after 3 months, and to 0.6 ± 0.0% after 4 months. Then, it decreased to 0.0 ± 0.0% at the end of storage. According to storage temperatures of 0, 25, 35, and 45 °C, after 8 months, it reached 0.16 ± 0.0%, 0.03 ± 0.0%, 0.0 ± 0.0%, and 0.0 ± 0.0%, respectively. Through a correlation analysis, it was found that sucrose had a negative (*p* < 0.05) correlation with storage period, storage temperature, color, moisture, HMF, DN, and FA and a positive (*p* < 0.05) correlation with fructose, glucose, EC, pH, and DN (Table 4).

**Table 3:** Sugar analysis of Talh *Acacia gerrardii* honey according to different temperatures and storage intervals for eight months from 2019–2020.

Parameters: (Codex Standards)	Temp. (°C)	Fresh Honey	Storage Interval (8 Months)								
			1	2	3	4	5	6	7	8	Mean
Fructose (31–42%)	0	38.7 ± 0.0 a	37.7 ± 0.0 b	37.3 ± 0.0 bc	37.1 ± 0.0 bc	37.2 ± 0.0 bc	37.0 ± 0.0 bc	37.3 ± 0.0 bc	36.7 ± 0.0 c	37.0 ± 0.0 c	37.0 ± 0.0 A
	25	38.7 ± 0.0 a	37.4 ± 0.0 b	37.4 ± 0.0 b	37.1 ± 0.0 bc	37.4 ± 0.0 b	37.4 ± 0.0 b	37.2 ± 0.0 bc	36.6 ± 0.0 cd	36.2 ± 0.0 d	37.0 ± 0.0 A
	35	38.7 ± 0.0 a	36.9 ± 0.0 b	36.5 ± 0.0 b	36.4 ± 0.0 bc	36.4 ± 0.0 bc	36.4 ± 0.0 bc	35.9 ± 0.0 c	35.2 ± 0.0 d	35.3 ± 0.0 d	36.0 ± 0.0 B
	45	38.7 ± 0.0 a	36.0 ± 0.0 b	35.3 ± 0.0 c	35.1 ± 0.0 c	35.0 ± 0.0 cd	34.4 ± 0.0 d	33.7 ± 0.0 e	33.3 ± 0.0 e	32.3 ± 0.0 f	35.0 ± 0.0 C
Glucose (23–32%)	0	30.8 ± 0.0 a	29.7 ± 0.0 b	29.2 ± 0.0 bc	28.7 ± 0.0 c	29.1 ± 0.0 bc	29.4 ± 0.0 bc	29.3 ± 0.0 bc	28.8 ± 0.0 bc	29.7 ± 0.0 b	29.0 ± 0.0 A
	25	30.8 ± 0.0 a	28.7 ± 0.0 b	28.7 ± 0.0 b	28.2 ± 0.0 bc	28.1 ± 0.0 bc	28 ± 0.0 bc	27.6 ± 0.0 cd	27 ± 0.0 d	27 ± 0.0 d	28.0 ± 0.0 B
	35	30.8 ± 0.0 a	27.2 ± 1.0 b	25.7 ± 0.0 bc	25.2 ± 1.0 cd	24.5 ± 1.0 cd	24.4 ± 1.0 cd	23.7 ± 1.0 d	23.6 ± 1.0 d	23.6 ± 1.0 d	25.0 ± 1.0 C
	45	30.8 ± 0.0 a	26.0 ± 1.0 b	23.7 ± 1.0 c	23.5 ± 1.0 c	23.3 ± 1.0 c	22.3 ± 1.0 cd	22.2 ± 1.0 cd	21.8 ± 1.0 cd	21.2 ± 1.0 d	24.0 ± 1.0 D
Reducing sugars (fructose and glucose) (≥60)	0	69.5 ± 0.3 a	67.4 ± 0.6 b	66.5 ± 0.3 bc	65.8 ± 0.3 bc	66.3 ± 0.3 bc	66.4 ± 0.6 bc	66.6 ± 0.3 bc	65.5 ± 0.3 bc	66.7 ± 0.4 bc	67.0 ± 0.2 A
	25	69.5 ± 0.3 a	66.1 ± 0.5 b	66.1 ± 0.5 b	65.3 ± 0.5 b	65.5 ± 0.3 b	65.4 ± 0.3 b	64.8 ± 0.3 b	63.6 ± 0.5 c	63.2 ± 0.8 d	66.0 ± 0.3 B
	35	69.5 ± 0.3 a	64.1 ± 1.0 b	62.2 ± 0.6 bc	61.6 ± 0.7 cd	61.9 ± 0.8 cd	60.8 ± 0.9 cde	59.6 ± 0.6 cde	58.8 ± 0.6 e	58.9 ± 0.7 e	62.0 ± 0.5 C
	45	69.5 ± 0.3 a	62.0 ± 1.0 b	59.0 ± 0.7 c	58.6 ± 0.8 c	58.3 ± 0.9 c	56.7 ± 0.7 cd	55.9 ± 0.6 de	55.1 ± 0.7 de	53.5 ± 0.9 e	59.0 ± 0.7 D
Sucrose (≤5%)	0	0.31 ± 0.0 bc	0.5 ± 0.0 ab	0.76 ± 0.0 a	0.45 ± 0.0 abc	0.6 ± 0.0 ab	0.6 ± 0.0 ab	0.4 ± 0.0 abc	0.2 ± 0.0 c	0.16 ± 0.0 c	0.5 ± 0.0 A
	25	0.31 ± 0.0 bc	0.35 ± 0.0 bc	0.48 ± 0.0 ab	0.23 ± 0.0 cd	0.61 ± 0.0 a	0.38 ± 0.0 abc	0.18 ± 0.0 cd	0.18 ± 0.0 cd	0.03 ± 0.0 d	0.3 ± 0.0 B
	35	0.31 ± 0.0 ab	0.23 ± 0.0 ab	0.3 ± 0.0 ab	0.2 ± 0.0 bc	0.5 ± 0.0 a	0.3 ± 0.0 ab	0.1 ± 0.0 bc	0.0 ± 0.0 c	0.0 ± 0.0 c	0.2 ± 0.0 C
	45	0.31 ± 0.0 ab	0.1 ± 0.0 bc	0.3 ± 0.0 bc	0.2 ± 0.0 bc	0.5 ± 0.0 a	0.2 ± 0.0 bc	0.2 ± 0.0 bc	0.01 ± 0.0 c	0.0 ± 0.0 c	0.2 ± 0.0 C

Means with the same letters are not significantly different from each other (*p* < 0.05, Duncan’s test). Lower case letters in table columns represent the comparison among values of individual sugar parameter of fresh honey, and honey stored at different temperature (0, 25, 35 and 45 °C) over a period of 1–8 months. Upper case letters in last column represent the comparison among mean values of individual sugar parameter after eight month’s storage of honey at four different temperatures.

**Table 4:** Pearson correlation coefficients between physicochemical characteristics of Talh *Acacia gerrardii* honey.

Variables	Color	Moisture	pH	EC	HMF	FA	DN	Fructose	Glucose	Sucrose
Storage Period	0.388 *	−0.025	−0.481 *	0.323 *	0.391 *	0.401 *	−0.240 *	−0.536 *	−0.432 *	−0.286 *
Storage Temp	0.450 *	0.087	−0.079 *	0.017	0.603 *	0.631 *	−0.423 *	−0.423 *	−0.636 *	−0.316 *
Color	1	−0.144 *	−0.711 *	−0.257 *	0.399 *	0.541 *	−0.498 *	−0.448 *	−0.549 *	−0.463 *
Moisture		1	−0.197 *	0.132 *	0.212 *	0.440 *	0.413 *	−0.235 *	−0.311 *	−0.105 *
pH			1	0.220 *	−0.822 *	−0.851 *	0.454 *	0.737 *	0.728 *	0.511 *
EC				1	0.111 *	0.155 *	0.013	−0.305 *	−0.305 *	0.241 *
HMF					1	0.852 *	−0.559 *	−0.842 *	−0.748 *	−0.272 *
FA						1	−0.309 *	−0.821 *	−0.892 *	−0.422 *
DN							1	0.456 *	0.402 *	−0.004 *
Fructose								1	0.868 *	0.240 *
Storage Period	0.388 *	−0.025	−0.481 *	0.323 *	0.391 *	0.401 *	−0.240 *	−0.536 *	−0.432 *	−0.286 *
Storage Temp	0.450 *	0.087	−0.079 *	0.017	0.603 *	0.631 *	−0.423 *	−0.423 *	−0.636 *	−0.316 *
Color	1	−0.144 *	−0.711 *	−0.257 *	0.399 *	0.541 *	−0.498 *	−0.448 *	−0.549 *	−0.463 *
Moisture		1	−0.197 *	0.132 *	0.212 *	0.440 *	0.413 *	−0.235 *	−0.311 *	−0.105 *
pH			1	0.220 *	−0.822 *	−0.851 *	0.454 *	0.737 *	0.728 *	0.511 *
EC				1	0.111 *	0.155 *	0.013	−0.305 *	−0.305 *	0.241 *

* Correlation is significant at the 0.05 probability level.

## 3. Discussion

The Talh tree, *A. gerrardii*, is one of the main pollen sources for honeybees in Saudi Arabia. Microscopic analyses of pollen can provide valuable information, such as the geographical source of honey, plant origin, and vegetation characteristics [24]. Numerous studies have reported that Talh honey from different regions of Saudi Arabia is monofloral with more than 40% acacia pollen grains [15,18,25]. This is in confirmation with our findings, where we found high pollen percentages of *A. gerrardii* in the tested honey samples. Monofloral honeys are highly preferred by consumers due to their phytochemical properties related to health issues [26]. Saudi Arabia is home to monofloral honeys from local plants [27]. The differences in pollen densities in the honey samples of three locations could be attributed to the diversity of vegetation in these different locations and possible foraging preferences of honeybees for different nectar resources [28,29].

Different factors such as bee species, geographic origin, plant type, climatic conditions, seasons, treatments, and storage conditions are associated with the chemical composition of honey [30]. The physicochemical parameters, especially the levels of FA, were investigated in this study. It was found that Talh honey had naturally occurring high levels of FA, which is in agreement with a previous study [17]. The FA value of newly harvested Talh honey was 93 ± 0.3 meq/kg, which is beyond the limits of the Codex Alimentarius Standard [13] and Gulf Standard Organization [20] for honey (≤50 meq/kg). Several studies have reported FA values that exceed standard limits in acacia honey collected from Saudi Arabia, Oman, Yemen, and Morocco [16,31,32,33,34]. The level of FA in honey provides useful information regarding its botanical origin [35]. FA normally varies based on floral origin, the presence of different organic acids, acids of bacterial origin, harvest season, and bee species [36,37]. Our results indicated that the high values of free acidity in Talh honey also increased during the storage period at all the temperatures. These results are in confirmation with previous studies [9,11,32,33,38,39]. This increasing tendency is attributed to the chemical changes and maturation processes of honey during storage, including the conversion of carbohydrates to alcohol and organic acids [40].

During the storage period, the progression of the FA level was affected by other parameters. This conclusion was drawn because FA showed a positive (*p* < 0.05) correlation with storage period, storage temperature, HMF, color, moisture, and EC, whereas FA was negatively (*p* < 0.05) correlated with DN, pH, and sugar content. Similar correlations for FA have been reported in previous studies [41,42,43,44]. It is worth noting that, if pH values decrease, then the acidity content of honey increases. The changes in FA and the negative correlation between FA and sugars could be explained by two reactions involving the enzymatic action of glucose-oxidase on glucose to produce gluconic acid [45] and the Millard reaction between amino acids and reducing sugars [46].

The moisture content in honey is dependent on environmental conditions such as temperature and relative humidity during honey production, flower nectar, harvesting season, processing techniques, and storage conditions [47,48]. In the present study, the initial moisture content of the Talh honey samples was 15 ± 0.1%, much lower than the maximum acceptable limit (20%) given by national and international standards [13,20]. The moisture content did not show a clear variation among fresh and stored honey [49]. We found only a slight decrease in moisture level during the storage period at different temperatures. Our findings are in line with previous findings where slight decreases in the moisture contents of all types of honey are recorded at the end of the storage times [9,50,51].

Furthermore, the current results showed that moisture content was positively correlated (*p* < 0.05) with EC, HMF, FA, and DN. However, moisture content was negatively correlated (*p* < 0.05) with storage period, pH, fructose, glucose, and sucrose. Similarly, several studies have shown moisture content of a significantly positive correlation with FA, EC, and DN [33,44]. In contrast, moisture content has been found to have significant negative correlations with HMF, carbohydrates, and pH [44,52]. The differences in correlations between moisture and HMF may result from differences in chemical properties, such as the pH and mineral contents of different honeys [41].

The results of this study classified the color of the examined Talh honey according to USDA-approved color standards from 1985 [21]. The color of honey usually ranges from light yellow to amber and dark amber to black in extreme cases, and it is sometimes even green or red [53]. The color of honey is one of the characteristics that indicates the plant source, and potential factors that might cause a color change include nectar and pollen, the age of honey frames, the Millard reaction, sugar caramelization, exposure to high temperature, prolonged storage, chlorophylls, carotenoids, flavonoids, and polyphenols [22,54,55]. In fact, darker honeys are favored for medicinal use due to their high contents of iron, manganese, copper, and phenolic compounds [22,56]. In the present study, Talh honey was classified as dark honey, which has been previously confirmed in several studies [16,53,57]. The results showed that the color value of Talh honey increased gradually toward darkness as the temperature and storage period increased. These findings are similar to those of previous studies [50,58]. Additionally, color changes were more pronounced at high temperatures, which is consistent with results that have been reported in previous studies [59,60,61].

In the current study, it was found that color was positively correlated (*p* < 0.05) with storage period, storage temperature, HMF, and FA. Color was negatively correlated (*p* < 0.05) with moisture, pH, EC, DN, fructose, and glucose. These results may indicate the impacts of these variables on the color of Talh honey. Some studies have reported that color was negatively correlated with storage period, storage temperature, EC, and pH [60] and positively correlated with EC, TSS, and pH [62]. A negative correlation for color, as found in present study, was reported with moisture content [62]. These contradictions in the analysis of color correlations indicate the strong effect of different chemical compositions of honeys from diverse origins. The physical properties of honey are greatly affected by continuously changing chemical compositions [63]. Additionally, honey appearance was changed according to extracting, handling, packing, and preserving methods [64].

The results showed electrical conductivity (EC) values higher than 0.8 mS/cm, which is the maximum limit indicated by Saudi [20] and international standards [13]. Similar results for EC were reported for Talh honey by Alqarni, et al. [16]. Usually, in comparison to dark-colored honey, lighter-colored honey indicates lower electrical conductivity [16,65]. The EC is closely associated with the concentration of mineral salts, organic acids, floral sources, storage time, and proteins in honey [16,22,66,67]. The current study showed a slight increase in the electrical conductivity values during the storage period. This result was previously confirmed by other studies [50].

The correlation analysis showed a positive correlation (*p* < 0.05) between EC and storage period, HMF, and FA, while a negative (*p* < 0.05) correlation was recorded between EC and fructose, glucose, and sucrose. Several studies have recorded negative correlations between EC and pH, moisture, FA, HMF, and sugars [43,44]. However, positive correlations of EC and FA and moisture were found [44]. Since EC is an indication of mineral content, which is highly dependent on the type of soil and botanical origin [68], as well as with compositional changes during storage [30], variations in the correlations of EC with other parameters are expected.

The pH of honey is of great importance during extraction and storage, as it influences honey texture, stability, and shelf life [34]. In this study, the pH values of Talh honey ranged from 4.0 ± 0.0 to 5.1 ± 0.1, which were within the recommended limits (pH 3.4 to 6.1) for fresh honey [13] and in accordance with those in previous reports [57,69]. Differences in pH depend on many factors, particularly geographical origin and climatic conditions [70]. Thus, pH can be considered a basic marker of the geographical origin of honey [71]. Temperature was the factor that most influenced the pH value, as a decrease in pH activity was observed in all the tested Talh honeys during the storage period, a result that is consistent with those of previous studies [48,50]. Evahelda, et al. [72] reported that, at the end of 12 weeks of storage, the pH values decreased to 3.89, 3.83, and 3.80 at storage temperatures of 20, 30, and 40 °C, respectively, from the first week of the storage process, which is consistent with our results. Similarly, acidic pH values (4.17 and 4.20) were recorded in Algerian and Saudi honeys [73,74]. In the present results, pH was positively (*p* < 0.05) correlated with EC, DN, fructose, glucose, and sucrose and negatively correlated with storage period, storage temperature, HMF, and FA. Similar negative correlations were reported for pH with HMF and FA [44]. However, Alyammahi [43] reported that pH had a positive correlation with moisture, FA, and sugars. These findings are not in complete accordance with the correlations reported in this study. This inconsistency may be explained by the fact that honey undergoes ongoing chemical reactions throughout the storage period. Acknowledging this fact is vital for determining honey behavior during storage [30]. In fact, pH correlations with some quality factors may indicate honey contamination. The pH value significantly increased if high-fructose corn syrup was added to honey [75].

HMF is found naturally in honey due to the action of normal honey acidity on reducing sugars. It is known to be a marker for determining honey freshness and deteriorating quality. HMF content tends to increase during processing or product aging. Thus, HMF is considered an indication of high temperature and poor storage conditions. In the international honey trade, the maximum permissible level of HMF is 40 mg/kg. However, honey from regions with tropical ambient temperatures should not exceed 80 mg [13]. There are many factors that influence HMF levels, such as temperature, storage conditions, source of flowers, and some chemical properties of honey, including pH, acidity, mineral content, and reducing sugars [61]. In present study, the mean value of the initial HMF content in the fresh Talh honey was 1 ± 0.2 mg/kg, and the HMF levels increased gradually during the storage period until they reached 281 ± 43.0 and 897 ± 22.5 mg/kg at 35 °C and 45 °C, respectively, after 8 months of storage. At 0 °C and 20 °C, the increases in HMF content did not exceed the acceptable limits during the complete storage period. Our results are in accordance with those of preceding reports on the levels of HMF in fresh honey, and these reports have shown that fresh honey contains very little or no HMF [27,76]. Our results are also in agreement with those of earlier studies conducted in Saudi Arabia, the United Arab Emirates, Oman, Yemen, and Pakistan [66,77]. It was also reported that honey with a low pH value produced more HMF during storage [78]. We found that the levels of HMF increased with an increase in the storage period, and the levels of HMF were not affected when stored at lower temperatures but were significantly elevated when stored at high temperatures. This is in confirmation with previous studies of different countries where HMF content is associated with storage period and temperature [39,41,61,73]. In Saudi Arabia, Alqarni, et al. [16] confirmed that some acacia samples had an HMF content higher than the permissible limit because they were stored for a longer period or were exposed to heat. The presence of HMF is an indication of a breakdown of sugar due to incorrect or long storage at high temperatures [79]. Positive correlations between HMF and storage period, storage temperature, moisture, color, and FA were found. Negative correlations were recorded between HMF and DN, pH, and sugar content. Similarly, other studies have reported that HMF is positively correlated with storage period and temperature [30], FA [42], and moisture [59] and negatively correlated with DN [80]. The chemical constituents of honey from different floral resources were correlated with the formation of HMF [46]. During storage, chemical changes in honey may affect nutritive and sensory features related to the chemical reactions involved in HMF formation [81].

Enzymatic activity is the basis for assessing honey quality [82]. Diastase is a vital enzyme introduced by bees during nectar conversion into honey [33]. In the present study, the diastase value of fresh Talh honey (16 ± 3.0 DN) was according to the range of international and national standards (≥8 DN). Likewise, Sajwani, et al. [33] illustrated that the diastase activity in 81% of acacia honey was over 8 DN, with mean of 12 DN on Gothe’s scale. Diastase content varies according to floral source, storage for long periods, geographical origins, and exposure to high temperatures [83]. Researchers observed that honey from warmer regions may have low diastase activity [84]. Serrano, et al. [85] found that the mean value of diastase was 20.48 ± 10.14 (range 3.99–49.42), expressed as a diastase number on Gothe’s scale. Our results showed that diastase activity decreased with increasing storage temperature and storage period: the diastase enzyme was more sensitive at 45 and 35 °C, while 0 and 25 °C had no significant effect on the enzyme during the storage period. These results are in agreement with those of Huang, et al. [82], who reported that the sensitivity of diastase activity decreased with increasing temperature and that the sensitivity of acacia honey was higher than that of other honey samples. Similar to our results, Moreira, et al. [81] showed a significant decrease in diastase activity in honey samples compared to that in fresh samples after storage at 35 and 40 °C. Qamer, et al. [38] reported similar results, with a decrease in diastase number in honey samples during storage for eight months. The same behavior of decreased diastase activity has also been noticed by different scientists [30,39,86].

The correlation analysis revealed that DN had a significantly positive correlation with moisture, pH, EC, fructose, and glucose, whereas it had a negative correlation with storage period, storage temperature, color, HMF, FA, and sucrose. Some findings have reported positive correlations between DN and EC and HMF [87,88], while other findings have shown negative correlations between DN and storage period and storage temperature [60,89], which is not in total agreement with the current study correlations. The discrepancy in correlation relationships between honey characteristics could be attributed to the effect of differences in floral and geographical origins and to the ongoing compositional changes in honey during storage time. With approximately 200 constituents, honey becomes a complex mixture, with chemical reactions such as oxidation, fermentation, and thermal processing responsible for these changes [68,90].

The three investigated sugars (fructose, glucose, and sucrose) decreased significantly during the storage period under all the temperatures. This declining trend for sugars was very clear at higher temperatures of 35 °C and 45 °C. The content of reducing sugars (fructose and glucose) in the fresh Talh honey samples was 69.5 ± 0.3%, which was in accordance with the national standards [20] and the international Codex Alimentarius standard [13]. The values of the reducing sugars obtained in this study were similar to those reported for acacia and other honeys from Oman, Saudi Arabia, Algeria, and Pakistan [27,66,77,91]. El Sohaimy, et al. [57] found that Saudi honey samples showed the highest value of reducing sugars (72.36 ± 0.32 g/100 g). The sucrose content was originally below standard limits (≤5%) in the fresh samples before storage. Therefore, while the sucrose content decreased during storage, it remained at acceptable levels. On the other hand, reducing sugars remained at acceptable levels only at 0 °C and 25 °C. This result implied the importance of maintaining honey quality under suitable temperatures not exceeding 25 °C.

In general, the sugar structure of honey is influenced by floral and geographical origins, climate, processing, and storage period [30,92]. Likewise, it has been noted that storing honey has a significant effect on honey sugars, as fructose and glucose contents decrease with increasing storage period [93,94]. Our results are consistent with previous findings, where reductions in monosaccharides are recorded compared with their original values before storage at temperatures of 10, 20, 30, and 40 °C [9,51,72]. A decrease in monosaccharides (fructose and glucose) in honey is expected due to the effects of heat and storage period, which leads to their degradation to HMF through the nonenzymatic Maillard reaction [46,95]. On the other hand, a decrease in disaccharides (sucrose) results from the enzymatic action of invertase, leading to the formation of monosaccharides [9,45,46]. Sucrose was detected at very low rates in fresh Talh honey, with values below 5% (0.31 ± 0.0%) ranging from 0 ± 0.0% to 0.76 ± 0.0%, which were within national and international standard limits. The sucrose values in the present study correspond to those of Algerian, Pakistani, and Romanian honeys, with reported sucrose contents of 1.79%, 2.11%, and 1.62%, respectively [96,97].

The correlation analysis for sugars in the Talh honey samples revealed positive correlations between sugars and pH and DN, and negative correlations between sugars and storage period, temperature, color, EC, HMF, FA, and moisture. These findings are in accordance with positive correlations between sugars and DN and pH [98,99] and negative correlations between sugars and moisture, AF, and EC [22,43,44].

## 4. Materials and Methods

The experiments were conducted at the Melittology and Honey Quality Research Laboratory at the Department of Plant Protection, King Saud University, Riyadh city, from 2019–2020. The physicochemical characteristics of honey samples were determined according to the recommended methods of the Association of Official Analytical Chemists [22,100,101] for free acidity (FA), pollen, color, moisture content, electrical conductivity (EC), pH value, hydroxymethylfurfural (HMF), diastase activity (DN), and sugar content.

### 4.1. Honey Samples

Ripened fresh honey samples were aseptically collected soon after harvest and extraction from three apiaries in three regions of Saudi Arabia (Asir in the south, Riyadh in the center, and Hail in the north) enriched with *Acacia gerrardii* Benth trees. The honey originating from *A. gerrardii* is locally named as Talh honey (TH) in Saudi Arabia. Each honey sample collected from the three regions was further divided into 12 aliquots of 500 g honey and was aseptically bottled. Four incubators with four different temperatures (0, 25, 35, and 45 °C) were maintained separately, and nine bottles (500 g each) of aliquots (three from each region) were stored in each incubator for eight months, representing Talh honey of Saudi Arabia. Fresh honey samples were analyzed upon arrival at the laboratory. Then, analyses were performed once every month throughout the storage period to determine the different phases of physicochemical parameters, as well as the effects of storage period and temperature on the properties of the honey, especially free acidity.

### 4.2. Melissopalynological Analysis

The botanical origins of the honey samples were confirmed according to an analysis of the honey pollen grains following the method recommended by the Association of Official Analytical Chemists for pollen, as well as the approaches of Louveaux, et al. [18] and Ohe, et al. [24]. Honey (10 g) was dissolved in 20 mL warm distilled water (40 °C) and centrifuged for 10 min at 2500 rpm. The solution was poured into a small tube and centrifuged again for 10 min. The entire sediment was placed on a slide, spread out over an area of 20 mm^2^, and dried by slightly heating to 40 °C. The sediment was mounted with glycerin gelatin and liquefied by heating in a water bath at 40 °C. The pollen grains present in the tested honey samples were identified with the aid of a pollen atlas [102]. Estimates of pollen grain frequencies in the honey samples were determined based on the system of Crane [103]: “Very frequent” for grains constituting more than 45%, “Frequent” for grains constituting 16–45%, “Rare” for grains constituting 3–15%, and “Sporadic” for grains constituting less than 3% of the total grains. Based on this analysis, all honey samples from the three regions were treated as one type of Saudi monofloral Talh honey.

### 4.3. Physicochemical Analysis

The main target for analysis in this study was free acidity. However, other characteristics of honey, such as moisture, color, electrical conductivity, pH, HMF content, sugar content, and diastase enzyme activity, were also determined for correlation purposes and a broader understanding of how storage conditions were reflected in these characteristics. The analysis was conducted following the AOAC guidelines [23,100,101]. Every sample was tested in triplicate for every parameter, and their mean values were obtained.

#### 4.3.1. Free Acidity (FA)

Free acidity and all other parameters were measured directly after extraction and monthly throughout the 8 months of the study for the stored honey under all four temperatures. The total number of samples analyzed during each analysis period was 36 (9 samples from 3 regions at 4 temperatures). The procedure for free acidity was performed by the titrimetric method: 10 g honey was dissolved in 75 mL deionized water. Then, the dissolved honey solution was titrated with sodium hydroxide (NaOH 0.05 N) until the pH value reached 8.5. Then, the final acidity number was obtained as meq/kg [100].

#### 4.3.2. Moisture Content

The refractometric method was used to determine the moisture content. In general, the refractive index increases with an increase in the solid content of a sample. The refractive indices of the honey samples were measured at ambient temperature using a refractometer (HAMMANN^®^ honey refractometer, Germany). The moisture content was measured in triplicate [100].

#### 4.3.3. Color

The color intensity of the honey samples was recorded according to the Pfund classifier. Briefly, homogeneous honey samples free of air bubbles were transferred into a cuvette in a 10 mm light bath until the cuvette was approximately half full. The cuvette was inserted into a color photometer (HI 96785, Hanna^®^ Instruments, Romania). Color grades were expressed in millimeters (0–150 mm), and Pfund grades were compared to an analytical-grade glycerol standard. Measurements were performed in triplicate for each sample using the approved color standards of the United States Department of Agriculture [21].

#### 4.3.4. Electrical Conductivity (EC)

The EC was determined using a Hanna^®^ pH PPM Meter (HI-9813-6N). The EC meter was first calibrated with deionized water, the conductance cell was dipped into a honey solution (10.0%), and the readings were recorded after stabilization of the instrument [101].

#### 4.3.5. pH

pH was measured using a Hanna^®^ pH PPM Meter (HI-9813-6N). Honey (10 g) was dissolved in 75 mL deionized water. The honey solution was transferred to a beaker, and when the meter obtained stable readings, they were recorded directly from the pH meter [101].

#### 4.3.6. Hydroxymethylfurfural (HMF)

HMF was determined according to the AOAC [100]. Five grams of honey was dissolved in 25 mL water and transferred quantitatively into a 50 mL volumetric flask. An amount of 0.5 mL Carrez I solution (15 g potassium ferrocyanide: K_4_[Fe(CN)_6_]·3H_2_O) was dissolved in DI water and diluted to 100 mL, and 0.5 mL Carrez II (30 g zinc acetate (Zn(CH3 CO2) 2 ∙2H2 O) was dissolved in DI water and diluted to 100 mL, and both were added and brought up to 50 mL with water. The solution was filtered through paper, rejecting the first 10 mL of the filtrate. Aliquots of 5 mL were placed in two test tubes: 5 mL DI water was added to one tube (sample solution), and 5 mL sodium bisulfite solution (0.2% in DI water) was added to the second tube (reference solution). The absorbance of the solutions at 284 and 336 nm was determined using a Thermo Scientific^®^ GENESYS 10S UV-Vis spectrophotometer. The HMF content was calculated using the following equation: HMF (mg/kg) = (A284) − (A336) × 149.7, where A284 is the absorbance at 284 nm, A336 is the absorbance at 336 nm, and 149.7 is a factor calculated by the molecular weight of HMF and the mass of the sample.

#### 4.3.7. Diastase Activity (DA)

Diastase activity was determined using 10 g honey, 5 mL acetate buffer, and 20 mL distilled water in a 50 mL beaker. When the sample was completely dissolved, 3 mL sodium chloride (0.5 M) was added to the solution. The solution was diluted to 50 mL with distilled water. Then, a starch solution was standardized using an iodine solution. Both solutions were fixed in a water bath previously set at 40 °C. Then, 5 mL of the starch solution was added to 10 mL of the honey solution. An aliquot was taken every 5 min and added to 10 mL iodine solution. The absorbance was recorded, and a calibration curve was obtained. The diastase activity was expressed as a diastase number (DN) according to [101,103].

#### 4.3.8. Sugar Content

The sugar profiles of the different honey samples were identified using high-performance liquid chromatography (HPLC) (Agilent Technologies^®^ HPLC with RID and carbohydrate column). The samples were prepared by adding 0.5 g honey to 20 mL distilled water. The solution was transferred to a 50 mL volumetric flask through filter paper. The volumetric flask was filled up to a 50 mL volume with water. Then, 1 mL honey solution was removed by pipette and added to a 2 mL HPLC vial. The sample was injected into the HPLC (Agilent Technologies^®^ HPLC with RID and carbohydrate column), and the chromatogram peaks of the sugars were analyzed by comparison with the peaks of standard sugars [104].

### 4.4. Statistical Analysis

The statistical analysis for the obtained values of all the parameters was performed using SAS^®^ 9.2 software. The results are expressed as the means ± SE. Duncan’s multiple range test was used to evaluate the significance of the differences (*p* < 0.05) between the results. A correlation analysis was performed between the parameters. 

## 5. Conclusions

Conclusively, fresh Talh honey has naturally high FA levels that are higher than standard limits, and this characteristic hinders the marketing of Talh honey in Saudi Arabia. It is, therefore, necessary to re-evaluate the Talh honey standard in the country. Postharvest handling temperatures (0, 25, 35, and 45 °C) and length of storage (1–8 months) had significant effects on the quality (physiochemical parameters: FA, HMF, RSs, and DA) of Talh honey originating from *Acacia gerrardii*. Honey stored at 0 and 25 °C maintained a quality comparable to that of fresh honey, and this is the recommended storage condition for honey. Honey storage at high temperatures (45 and 35 °C) resulted in the deterioration of the quality of the honey, which started by increasing the FA and EC levels after one month and increased gradually with the passage of storage time. The storage of honey at higher temperatures altered its physiochemical parameters (FA, HMF, DA, and RSs) quickly and significantly during short storage durations, ranging from 1–2 months for 45 °C and 2–7 months for 35 °C. Certain physiochemical parameters, such as moisture content, pH, color, and sucrose, were not affected by storage conditions. It is, therefore, recommended that honey should be stored at temperatures not exceeding 25 °C to maintain the quality of honey. Thus, this study can be very beneficial for educating beekeepers on honey postharvest storage conditions that can alleviate unseen damage to honey quality and maintain the freshness of honey for better marketing.

## Figures and Tables

**Table 1:** *Acacia gerrardii* pollen grains in Talh honey dependent on the analysis of the honey pollen grains.

No.	Type of Honey	Geographical Origin of Honey	Detail of Pollens (%)	*Acacia gerrardii* % Pollen Grains (Range)	Mean (%)
1	Talh honey	Riyadh	Fabaceae (Talh) 88% Rhamnaceae (Zizphus) 8% Asteraceae and others 4%	88 (82–93)	69
2	Talh honey	Asir	Fabaceae (Talh) 64% Rhamnaceae (Zizphus) 20% Brassicaceae 11% Asteraceae and others 5%	64 (62–66)	
3	Talh honey	Hail	Fabaceae (Talh) 54% Amaranthaceae 31% Poaceae 5% Malvaceae and others 10%	54 (53–55)	

## Data Availability

Data are available on request due to privacy restrictions.

## Supplementary Materials

The following supporting information can be downloaded at: https://www.mdpi.com/article/10.3390/molecules27185959/s1, Table S1: Comparison of physicochemical parameters in Talh honey (originating from *Acacia gerrardii)* from different locations according to different temperatures and storage intervals for eight months; Table S2: Comparison of sugar contents in Talh *Acacia gerrardii* honey from different locations according to different temperatures and storage intervals; Table S3: Physicochemical parameters in Talh honey collected from Riyadh region and stored at different temperatures and storage for eight months; Table S4: Comparison of physicochemical parameters in Talh honey collected from Asir region and stored at different temperatures and storage for eight months; Table S5. Comparison of physicochemical parameters in Talh honey collected from Hail region and stored at different temperatures and storage for eight months; Table S6: Comparison of sugar contents in Talh Acacia gerrardii honey collected from Riyadh region and stored at different temperatures and storage interval; Table S7: Comparison of sugar contents in Talh Acacia gerrardii honey collected from Asir region and stored at different temperatures and storage interval; Table S8: Comparison of sugar contents in Talh Acacia gerrardii honey collected from Hail region and stored at different temperatures and storage interval.
