# Supplementary material for: Impact of Different Storage Regimes on the Levels of Physicochemical Characteristics, Especially Free Acidity in Talh (Acacia gerrardii Benth.) Honey
Source: Molecules. 2022 Sep 13;27(18):5959. doi: 10.3390/molecules27185959 (PMC9505800; doi:10.3390/molecules27185959)
Supplement: Supplementary file 1 [file molecules-27-05959-s001.zip › molecules-1801144-supplementary.pdf]

Supplementary Table

Table S1. Comparison of mean values of physicochemical parameters in Talh honey (orignated from Acacia gerrardii) of different locations according to different temperatures and storage for eight months

Parameters: (Codex standards)	Temp. (°C)	Mean (after 8 months) Riyadh	Asir	Hail	Combined Mean of values in a row
Moisture (≤20%)	0	15 a	15.2 b	14.7 b	15±0.1 AB
	25	14.6 c	15.2 b	14.5 c	14.8±0.1 C
	35	14.7 b	15.4 a	14.7 b	14.9±0.1 B
	45	15 a	15.4 a	14.8 a	15.1±0.1 A
Color Pfund (0-150 mm)*	0	114 c	127 c	150 a	130±5.0 A
	25	128 b	136 b	150 a	138±5.0 A
	35	145 a	145 a	150 a	147±4.0 B
	45	145 a	146 a	150 a	147±4.0 C
EC (mS/cm) (≤0.8)	0	1.73 c	1.58 ab	1.38 a	1.57±0.1 A
	25	1.77 c	1.58 ab	1.39 a	1.58±0.1 A
	35	1.75 b	1.57 b	1.38 a	1.57±0.1 A
	45	1.75 b	1.59 a	1.39 a	1.58±0.1 A
pH (3.4-6.1)	0	5.2 a	4.8 a	4.7 a	4.9±0.1 A
	25	5.1 b	4.8 a	4.7 a	4.9±0.1 A
	35	4.9 c	4.6 b	4.6 b	4.7±0.1 B
	45	4.5 d	4.3 c	4.4 c	4.4±0.1 C
Free acidity (meq/kg) (≤50)	0	83 d	103 c	96 c	94±0.3 D
	25	92 c	108 c	97 c	99±0.3 C
	35	111 b	125 b	106 b	114±0.5 B
	45	131 a	149 a	120 a	133±0.8 A
HMF (mg/kg) (≤40)**	0	1.5 c	0.7 c	2.29 c	2±0.4 C
	25	6.9 c	1.7 c	16.36 c	8±3.0 C
	35	90 b	113.8 b	239.86 b	148±40.8 B
	45	504 a	499.1 a	397.27 a	467±99.7 A
Diastase activity (DN) (≥8)	0	10.8 a	28 a	7.5 a	15±3.0 A
	25	11.3 a	25 b	6.60 b	14±3.0 A
	35	6.1 b	19 c	4.99 c	10±2.0 B
	45	3 c	8 d	3.13 d	5.0±2.0 C

Means with the same letters are not significantly different from each other ($P < 0.05$, Duncan's test).
*The color was determined in (mm) on the Pfund scale according to U.S. Department of Agriculture classifications. ** in tropical regions (80 mg/kg)

Table S2 : Comparison of mean values of sugar contents in Talh *Acacia gerrardii* honey of different locations according to different temperatures and storage interval

**Parameters:** (Codex standards)	**Temp. (°C)**	**Mean (after 8 months)** Riyadh	**Asir**	**Hail**	**Combined Mean of values in a row**
Fructose (31-42%)	0	37 a	37.2 a	37.6 a	37.0±0.0 A
	25	37 a	37.3 a	37.5 a	37.0±0.0 A
	35	36 b	36.1 b	36.7 b	36.0±0.0 B
	45	35 c	34.6 c	35.3 c	35.0±0.0 C
Glucose (23-32%)	0	30 a	29.3 a	29.2 a	29.0±0.0 A
	25	28 b	27.7 b	29.1 a	28.0±0.0 B
	35	25 c	24 c	27.2 b	25.0±1.0 C
	45	24 d	22.3 d	25.4 c	24.0±1.0 D
Reducing sugars (Fructose +Glucose) (≥60)	0	67 a	66.5 a	66.8 a	67.0±0.2 A
	25	65 b	65 b	66.6 b	66.0±0.3 B
	35	61c	60.1c	62.5 c	62.0±0.5 C
	45	59 d	56.9 d	60.7 d	59.0±0.7 D
Sucrose (≤5%)	0	0.8 a	0.2 a	0.4 a	0.5±0.0 A
	25	0.5b	0.2 a	0.2 b	0.3±0.0 B
	35	0.4 c	0.2 a	0.1 b	0.2±0.0 C
	45	0.3 c	0.2 a	0.2 b	0.2±0.0C

Means with the same letters are not significantly different from each other ($P < 0.05$, Duncan's test).

Table S3. Physicochemical parameters in Talh honey collected from Riyadh region and stored at different temperatures and storage for eight months

Parameters: (Codex standards)	Temp. (°C)	Fresh honey (Riyadh)	Storage interval (8 months)								
			1	2	3	4	5	6	7	8	Mean
Moisture (≤20%)	0	15	15	15	15	15	15	15	14.9	14.9	15 a
	25	15	14.6	14.5	14.5	14.5	14.5	14.5	14.5	14.4	14.6 c
	35	15	14.6	14.6	14.6	14.6	14.8	14.8	14.9	14.9	14.7 b
	45	15	14.9	14.9	15	15	15	14.9	14.9	14.9	15 a
Color Pfund (0-150 mm)*	0	104	105	107	108	112	119	121	122	125	114 c
	25	104	111	119	124	127	133	136	145	150	128 b
	35	104	150	150	150	150	150	150	150	150	145 a
	45	104	150	150	150	150	150	150	150	150	145 a
EC (mS/cm) (⩽0.8)	0	1.6	1.7	1.7	1.7	1.7	1.8	1.8	1.8	1.8	1.73 c
	25	1.6	1.7	1.7	1.7	1.8	1.8	1.8	1.8	1.8	1.77 c
	35	1.6	1.7	1.7	1.7	1.8	1.8	1.8	1.8	1.8	1.75 b
	45	1.6	1.8	1.7	1.7	1.8	1.8	1.8	1.8	1.8	1.75 b
pH (3.4-6.1)	0	5.3	5.3	5.3	5.3	5.2	5.2	5.2	5.2	5.1	5.2 a
	25	5.3	5.1	5.1	5.1	5.1	5.1	5.1	5.0	5.0	5.1 b
	35	5.3	5.0	5.0	4.9	4.9	4.8	4.7	4.6	4.6	4.9 c
	45	5.3	4.8	4.7	4.6	4.5	4.3	4.3	4.2	4.1	4.5 d
Free acidity (meq/kg) (⩽50)	0	82	82	82	82	83	83	83	83	83	83 d
	25	82	83	85	88	94	96	100	102	102	92 c
	35	82	91	103	112	116	119	122	125	126	111 b
	45	82	106	118	125	134	144	151	160	163	131 a
HMF (mg/kg) (⩽40)**	0	0.2	1.2	1.7	1.8	1.8	1.8	1.6	1.9	1.6	1.5 c
	25	0.2	3.6	6.1	6.9	7.8	8.3	8.8	10.3	10.4	6.9 c
	35	0.2	15.8	24	47.3	76.2	101.3	167	183.3	194.7	90 b
	45	0.2	112.3	244.8	348	614	701.7	754.3	834.7	933	504 a
Diastase activity (DN) (⩾8)	0	12.3	10.6	10.6	10.5	10.5	10.5	10.5	10.5	10.8	10.8 a
	25	12.3	12.3	11.9	11.4	11.4	11	10.8	10.7	10.1	11.3 a
	35	12.3	9	6.3	5.4	5.1	4.9	4.2	3.9	3.6	6.1 b
	45	12.3	5.7	3.9	2.1	1.5	1	0.5	0	0	3 c

Means with the same letters are not significantly different from each other ($P < 0.05$, Duncan's test).

*The color was determined in (mm) on the Pfund scale according to U.S. Department of Agriculture classifications. ** in tropical regions (80 mg/kg).

Table S4. Comparison of physicochemical parameters in Talh honey collected from Asir region and stored at different temperatures and storage for eight months

Parameters: (Codex standards)	Temp. (°C)	Fresh honey (Asir)	Storage interval (8 months)								
			1	2	3	4	5	6	7	8	Mean
Moisture (≤20%)	0	15.2	15.2	15.3	15.3	15.3	15.2	15.2	15.2	15.2	15.2 b
	25	15.2	15.2	15.2	15.2	15.2	15.2	15.2	15.2	15.2	15.2 b
	35	15.2	15.2	15.2	15.4	15.5	15.5	15.5	15.5	15.5	15.4 a
	45	15.2	15.3	15.3	15.4	15.5	15.5	15.5	15.5	15.5	15.4 a
Color Pfund (0-150 mm)*	0	115	121	121	123	126	130	136	136	136	127 c
	25	115	121	130	131	134	142	148	150	150	136 b
	35	115	143	150	150	150	150	150	150	150	145 a
	45	115	150	150	150	150	150	150	150	150	146 a
EC (mS/cm) (≤0.8)	0	1.43	1.53	1.55	1.53	1.64	1.64	1.63	1.64	1.64	1.58 ab
	25	1.43	1.53	1.54	1.54	1.65	1.61	1.62	1.63	1.64	1.58 ab
	35	1.43	1.53	1.54	1.54	1.64	1.60	1.63	1.63	1.63	1.57 b
	45	1.43	1.55	1.53	1.52	1.64	1.66	1.65	1.65	1.66	1.59 a
pH (3.4-6.1)	0	5	4.9	4.8	4.8	4.8	4.8	4.8	4.8	4.8	4.8 a
	25	5	4.9	4.8	4.8	4.8	4.8	4.8	4.8	4.8	4.8 a
	35	5	4.9	4.8	4.7	4.6	4.5	4.4	4.4	4.3	4.6 b
	45	5	4.7	4.6	4.4	4.3	4.1	4.1	4.0	3.9	4.3 c
Free acidity (meq/kg) (≤50)	0	102	102	103	104	104	104	104	104	104	103 c
	25	102	103	106	109	110	110	110	110	113	108 c
	35	102	113	119	124	126	131	133	138	139	125 b
	45	102	125	138	146	155	164	166	169	174	149 a
HMF (mg/kg) (≤40)**	0	0.3	0.7	0.7	0.7	0.7	0.7	0.8	0.8	0.9	0.7 c
	25	0.3	1.3	1.1	1.8	1.8	2	2.1	2.4	2.5	1.7 c
	35	0.3	1.4	64	91.7	141.7	165.3	176.7	188.3	195	113.8 b
	45	0.3	164	338	445	493.3	583.3	715	810	943.3	499.1 a
Diastase activity (DN) (≥8)	0	29	28	28	28	28	28	28	28	28	28 a
	25	29	27	26	25	24	24	23	23	22	25 b
	35	29	28	20	19	17	16	15	15	13	19 c
	45	29	16	8	6	3	2	2	1	1	8 d

Means with the same letters are not significantly different from each other ($P < 0.05$, Duncan's test).

*The color was determined in (mm) on the Pfund scale according to U.S. Department of Agriculture classifications. ** in tropical regions (80 mg/kg).

Table S5. Comparison of physicochemical parameters in Talh honey collected from Hail region and stored at different temperatures and storage for eight months

Parameters: (Codex standards)	Temp. (°C)	Fresh honey (Hail)	Storage interval (8 months)								
			1	2	3	4	5	6	7	8	Mean
Moisture (≤20%)	0	14.7	1.47	14.7	14.7	14.7	14.7	14.6	14.6	14.6	14.7 b
	25	14.7	14.7	14.6	14.6	14.4	14.4	14.3	14.3	14.3	14.5 c
	35	14.7	14.7	14.7	14.7	14.7	14.7	14.7	14.7	14.7	14.7 b
	45	14.7	14.8	14.8	14.8	14.8	14.8	14.8	14.8	14.8	14.8 a
Color Pfund (0-150 mm)*	0	150	150	150	150	150	150	150	150	150	150 a
	25	150	150	150	150	150	150	150	150	150	150 a
	35	150	150	150	150	150	150	150	150	150	150 a
	45	150	150	150	150	150	150	150	150	150	150 a
EC (mS/cm) (≤0.8)	0	1.32	1.33	1.33	1.41	1.40	1.42	1.42	1.42	1.42	1.38 a
	25	1.32	1.36	1.33	1.33	1.41	1.43	1.44	1.43	1.43	1.39 a
	35	1.32	1.34	1.34	1.34	1.41	1.40	1.42	1.42	1.42	1.38 a
	45	1.32	1.39	1.40	1.32	1.40	1.40	1.43	1.44	1.43	1.39 a
pH (3.4-6.1)	0	4.9	4.7	4.7	4.7	4.7	4.7	4.7	4.7	4.7	4.7 a
	25	4.9	4.7	4.7	4.7	4.7	4.7	4.7	4.6	4.6	4.7 a
	35	4.9	4.7	4.7	4.6	4.6	4.5	4.4	4.4	4.3	4.6 b
	45	4.9	4.6	4.5	4.4	4.3	4.2	4.2	4.0	4.0	4.4 c
Free acidity (meq/kg) (≤50)	0	96	96	96	96	96	96	96	96	96	96 c
	25	96	96	96	97	97	98	98	98	99	97 c
	35	96	101	102	103	104	110	111	113	116	106 b
	45	96	104	113	117	120	127	131	134	139	120 a
HMF (mg/kg) (≤40)**	0	1.23	1.27	1.20	1.30	2.57	2.60	3.5	3.5	3.47	2.29 c
	25	1.23	5.37	5.33	11.47	17.67	20.67	25.33	27	33.17	16.36 c
	35	1.23	40.7	180.6	187.5	230	302.7	360	396	452	239.86 b
	45	1.23	104	197.3	273.6	315.7	498.7	663.3	706	815	397.27 a
Diastase activity (DN) (≥8)	0	8	7.9	7.6	7.4	7.4	7.5	7.3	7.3	7.1	7.5 a
	25	8	7.26	6.80	6.45	6.50	6.27	6.30	6.07	5.83	6.60 b
	35	8	6.36	5.83	5.11	4.89	4.20	3.83	3.58	3.13	4.99 c
	45	8	4.32	3.70	3.47	2.83	2.27	1.90	1.73	0.0	3.13 d

Means with the same letters are not significantly different from each other ($P < 0.05$, Duncan's test).

*The color was determined in (mm) on the Pfund scale according to U.S. Department of Agriculture classifications. ** in tropical regions (80 mg/kg).

Table S6 : Comparison of sugar contents in Talh *Acacia gerrardii* honey collected from Riyadh region and stored at different temperatures and storage interval

**Parameters:** (Codex standards)	**Temp.** (°C)	**Fresh honey (Riyadh)**	**Storage interval (8 months)**								
			**1**	**2**	**3**	**4**	**5**	**6**	**7**	**8**	**Mean**
Fructose (31-42%)	0	39	38	37	37	37	37	37	37	37	37 a
	25	39	37	37	37	37	37	37	37	36	37 a
	35	39	38	36	36	36	36	36	36	36	36 b
	45	39	37	35	34	34	34	33	33	32	35 c
Glucose (23-32%)	0	31	31	30	29	29	29	29	29	29	30 a
	25	31	28	28	28	28	28	27	27	26	28 b
	35	31	30	26	24	24	24	23	23	22	25 c
	45	31	30	23	22	22	23	22	21	21	24 d
Reducing sugars (Fructose +Glucose) (≥60)	0	70	69	67	66	66	66	66	66	66	67 a
	25	70	65	65	65	65	65	64	64	62	65 b
	35	70	68	62	60	60	60	59	59	58	62 c
	45	70	67	58	56	56	57	55	54	53	58 d
Sucrose (≤5%)	0	0.6	0.8	1.4	1	1	1	0.9	0.2	0.5	0.8 a
	25	0.6	0.5	0.9	0.4	1	0.6	0.3	0.1	0.1	0.5 b
	35	0.6	0.3	0.6	0.1	0.9	0.7	1	0	0	0.4 c
	45	0.6	0	0.5	0.1	0.9	0.3	0.1	0	0	0.3 c

Means with the same letters are not significantly different from each other ($P < 0.05$, Duncan's test).

Table S7 : Comparison of sugar contents in Talh *Acacia gerrardii* honey collected from Asir region and stored at different temperatures and storage interval

**Parameters:** (Codex standards)	**Temp.** (°C)	**Fresh honey (Asir)**	**Storage interval (8 months)**								
			**1**	**2**	**3**	**4**	**5**	**6**	**7**	**8**	**Mean**
Fructose (31-42%)	0	38.5	37.3	37.3	37.0	37.3	37.3	37.3	36.0	37.0	37.2 a
	25	38.5	37.7	37.7	37.0	37.7	37.7	37.7	36.0	36.0	37.3 a
	35	38.5	36.3	36.3	36.3	36	36	35.7	35	35	36.1 b
	45	38.5	35	35	35	34.7	34	34	33	32	34.6 c
Glucose (23-32%)	0	30.7	29	29	28.7	29	30	29.7	28.3	29.7	29.3 a
	25	30.7	28.7	28.7	27.7	27.7	27.7	26.7	26	26	27.7 b
	35	30.7	24.3	24.3	24.3	23.7	22.3	22	22	22	24 c
	45	30.7	22.7	22.7	22.7	22	20.3	20.3	20.7	19	22.3 d
Reducing sugars (Fructose +Glucose) (≥60)	0	69.2	66.3	66.3	65.7	66.3	67.3	67	64.3	66.7	66.6 a
	25	69.2	66.4	66.4	64.7	65.4	65.4	64.4	62	62	65.1 b
	35	69.2	60.6	60.6	60.6	59.7	58.3	57.7	57	57	60.1 c
	45	69.2	57.7	57.7	57.7	56.7	54.3	54.3	53.7	51	56.9 d
Sucrose (≤5%)	0	0.1	0.3	0.3	0.1	0.3	0.3	0.2	0.1	0.0	0.2 a
	25	0.1	0.4	0.4	0.1	0.4	0.2	0.1	0.3	0.0	0.2 a
	35	0.1	0.3	0.3	0.3	0.2	0.1	0.0	0.0	0.0	0.2 a
	45	0.1	0.3	0.3	0.3	0.3	0.0	0.1	0.0	0.0	0.2 a

Means with the same letters are not significantly different from each other ($P < 0.05$, Duncan's test).

Table S8 : Comparison of sugar contents in Talh *Acacia gerrardii* honey collected from Hail region and stored at different temperatures and storage interval

**Parameters:** (Codex standards)	**Temp.** (°C)	**Fresh honey (Hail)**	**Storage interval (8 months)**								
			**1**	**2**	**3**	**4**	**5**	**6**	**7**	**8**	**Mean**
Fructose (31-42%)	0	38.5	37.7	37.7	37.7	37.7	37.7	37.7	37.0	37.3	37.6 a
	25	38.5	37.7	37.7	37.7	37.7	37.7	37.0	36.9	37.0	37.5 a
	35	38.5	37.0	37.0	37.0	37.2	37.3	36.0	35.0	35.3	36.7 b
	45	38.5	36.0	36.0	36.0	35.9	35.0	34.0	33.0	32.7	35.3 c
Glucose (23-32%)	0	30.2	29.0	29.0	28.5	29.0	29.0	29.0	29.0	30.0	29.2 a
	25	30.2	29.3	29.3	29.3	29.0	28.7	29.0	28.0	29.0	29.1 a
	35	30.2	27.4	27.4	27.4	27.4	27.0	26.0	26.0	26.3	27.2 b
	45	30.2	25.7	25.7	25.7	25.7	24.0	24.0	24.0	24.0	25.4 c
Reducing sugars (Fructose +Glucose) (≥60)	0	68.7	66.7	66.7	66.2	66.7	66.7	66.7	66	67.3	66.9 a
	25	68.7	67	67	67	66.7	66.4	66	64.9	66	66.6 a
	35	68.7	64.4	64.4	64.4	64.6	64.3	62	61	61.6	63.9 b
	45	68.7	61.7	61.7	61.7	61.6	59	58	57	56.7	60.7 c
Sucrose (≤5%)	0	0.3	0.6	0.6	0.3	0.6	0.6	0.2	0.3	0.0	0.4 a
	25	0.3	0.2	0.2	0.2	0.5	0.4	0.2	0.1	0.0	0.2 b
	35	0.3	0.1	0.1	0.1	0.3	0.2	0.2	0.0	0.0	0.1 b
	45	0.3	0.1	0.1	0.1	0.3	0.4	0.4	0.0	0.0	0.2 b

Means with the same letters are not significantly different from each other ($P < 0.05$, Duncan's test).
